# Editorial: Soil microbiome community and functional succession mechanism driven by different factors in agricultural ecology

**DOI:** 10.3389/fmicb.2023.1276119

**Published:** 2023-08-29

**Authors:** Bin Huang, Wensheng Fang, Qin Gu, Bruno Tilocca

**Affiliations:** ^1^Tobacco Research Institute, Chinese Academy of Agricultural Sciences, Qingdao, China; ^2^Institute of Plant Protection, Chinese Academy of Agricultural Sciences, Beijing, China; ^3^Department of Entomology, Faculty of Plant Science, College of Plant Protection, Nanjing Agricultural University, Nanjing, China; ^4^Department of Health Sciences, University Magna Graecia of Catanzaro, Catanzaro, Italy

**Keywords:** agroecosystem, microbiome, community assembly, functional diversity, driving factor

In agroecosystems, soil microbes play an important role in soil nutrient conversion, plant growth regulation, soil-borne disease prevention and control, and degradation of harmful substances, and are an important part of soil microecological health (Kumar and Verma, 2019). Soil microbial community composition and function were significantly affected and changed by various agricultural operations and human factors in the long or short term, such as planting different crops (Zhang et al., 2013), applying pesticides (Sim et al., 2022), applying fertilizers (Bebber and Richards, 2022) and different field management (Guo et al., 2022). These agricultural operations may have positive effects on crop growth or pest control, but long-term effects may decrease the soil microbial diversity and stability, resulting in soil microecological imbalance. Tosi et al. (2021) showed that long-term (10 years) application of nitrogen fertilizer significantly increased soil bacterial biomass but decreased fungal abundance, and this change in microbial structure may lead to changes in nutrient cycling and crop growth. Shi et al. (2021) indicated that long-term continuous cultivation of edible lily resulted in the accumulation of pathogenic microbes *Streptomyces* and *Bacillus simplex*, while the decrease of *Sphingomonas and Hyphomicrobium* that were beneficial to plants was the cause of consecutive replanting problem. Therefore, the study and evaluation of soil microbiome community structure and functional succession under various abiotic factors (agricultural measures such as field management, pesticide spraying and fertilization) and biological factors (different crops and biocontrol microorganisms, etc.) are crucial for soil remediation and improvement, soil-borne disease control, plant growth and development, and soil health (Wen et al., 2023; Yang et al., 2023). However, most current studies have focused on the effects of various factors on the abundance and community structure of farmland soil microbes, while few studies have focused on the key drivers and potential mechanisms of soil microbial changes, as well as the interactions between the microbiome and crop roots and pathogenic microbes.

This Research Topic focuses on microbial assembly, community succession, and functional changes driven by different factors, and reveals the main drivers and potential mechanisms affecting the structure and function of soil microbial communities. Among the 42 research papers included, it was mainly reported that abiotic factors such as field cultivation management (five papers), pesticide spraying (six papers) and fertilization (10 papers) and biological factors (10 papers) changed soil microbial community structure and function, and such changes mainly affected crop growth and yield, disease occurrence and nutrient conversion. Among them, there are 13 articles analyzing the plant rhizosphere soil microbial community structure and function. In addition, 20 papers have analyzed the main influencing factors and potential mechanisms that cause changes in microbial communities.

Several original research papers published in this Research Topic focus on locations and crop genotype on the rhizosphere microbial community structure and functions. In this sense, the findings reported by Zhang et al. confirmed that locations affected the microbial community greater than that of tea varieties, and fungi might be more sensitive to the change in microenvironments. The abundance of arbuscular mycorrhizal fungi (AMF) was higher in Zhongcha 108 than that in Longjing 43. Field experiments further confirmed that the colonization rate of AMF was higher in Zhongcha 108. This finding testified that AMF could be the major beneficial tea rhizosphere microbes that potentially function in enhanced disease resistance.

At the same time, the findings from Yang et al. suggested that soil origin and cotton genotype modulated microbiome assembly with soil predominantly shaping rhizosphere microbiome assembly, while host genotype slightly tuned this recruitment process by changing the abundance of specific microbial consortia. Their results revealed that soil origin was the primary factor causing divergence in rhizosphere microbial community, with plant genotype playing a secondary role.

He et al. described evidence that the application of herbicide significantly impacted soil bacterial network composition. Both low and high doses of clomazone decreased bacterial network nodes, links, and average degrees as well as the network modules and stability. Overall, the results suggest that soil bacterial species connections, modularization, and network stability were significantly impacted by clomazone.

Li et al. indicated that soybean continuous changed soil microbial community by decreasing bacterial richness index while increasing fungal richness index. The plant pathogen fungi *Lectera* sp., *Plectosphaerella* sp., and *Volutella* sp. increased with continuous cropping years which lead to soybean yield seriously reduced.

Bai et al. found that long-term cultivation significantly alters soil properties and bacterial communities. Compared with grassland, cultivated land decreased soil pH, SOC and TN, and significantly increased soil EC, P and K, and soil properties varied significantly with different cultivation years. Grassland reclamation increases the diversity of bacterial communities, the relative abundance of Proteobacteria decreased and the relative abundance of Chloroflexi and Nitrospirae increased with the increase of cultivated land years.

Zhan et al. tested the effects of nitrogen addition and plant litter manipulation on soil fungal and bacterial communities in a semiarid sandy land. The results revealed that nitrogen addition significantly decreased soil microbial alpha diversity. N addition and litter manipulation had significantly interactive influences on soil microbial beta diversity, and litter manipulation had significantly decreased soil microbial beta diversity (*p* < 0.05) in the case of nitrogen addition. These results provide evidence that plant and soil microbial community respond differently to the treatments of N addition and litter manipulation.

Several other research articles focused on discovering the microbial response mechanism after exposure to different level and type fertilizer. Wang et al. reported that fertilization levels had significant effects on rhizosphere microorganisms. Cucumber could maintain microbial communities by secreting beneficial metabolites under slight excessive fertilization, but under extremely excessive fertilization, the self-regulating ability of cucumber plants and rhizosphere soil was insufficient to cope with high salt stress. The study provided new insights into the interaction of plant rhizosphere soil metabolites and soil microbiomes under the different fertilization levels. Niu et al. displayed that the application of slow-release fertilizers and reduced fertilizer application could improve soil physical and chemical properties as well as soil microbial community structure and diversity, contributing to sustainable soil development.

In summary, together the articles in this Research Topic make a significant contribution to soil microbial community characteristics following exposure to a series of farm operations such as pesticide application, crop genotypes, continuous cropping, long-term tillage, and over-fertilization. The obtained knowledges from this topic as a step toward a better comprehension of microbial community assembly and succession in agricultural production system.

## Author contributions

BH: Writing—original draft, Writing—review and editing. BT: Writing—review and editing. QG: Writing—review and editing. WF: Supervision, Writing—original draft, Writing—review and editing.

## References

[B1] Bebber D. P. Richards V. R. (2022). A meta-analysis of the effect of organic and mineral fertilizers on soil microbial diversity. Appl. Soil Ecol. 175, 104450. 10.1016/j.apsoil.2022.104450

[B2] Guo Z. Liu C. Hua K. Wang D. Wan S. He C.. (2022). Temporal variation of management effects on soil microbial communities. Geoderma 418, 115828. 10.1016/j.geoderma.2022.115828

[B3] Kumar A. Verma J. P. (2019). “The Role of Microbes to Improve Crop Productivity and Soil Health,” i*n Ecological Wisdom Inspired Restoration Engineering*, eds V. Achal, A. Mukherjee (Singapore: Springer), 249–265.

[B4] Shi G. Sun H. Calderón-Urrea A. Li M. Yang H. Wang W.. (2021). Bacterial communities as indicators for soil health under continuous cropping system. Land Degrad. Dev. 32, 2393–2408. 10.1002/ldr.391927699437

[B5] Sim J. Doolette C. Vasileiadis S. Drigo B. Wyrsch E. R. Djordjevic S. P.. (2022). Pesticide effects on nitrogen cycle related microbial functions and community composition. Sci. Total Environ. 807, 150734. 10.1016/j.scitotenv.2021.150734 34606862

[B6] Tosi M. Deen W. Drijber R. McPherson M. Stengel A. Dunfield K.. (2021). Long-term N inputs shape microbial communities more strongly than current-year inputs in soils under 10-year continuous corn cropping. Soil Biol. Biochem. 160, 108361. 10.1016/j.soilbio.2021.108361

[B7] Wen T. Xie P. Liu H. Liu T. Zhao M. Yang S.. (2023). Tapping the rhizosphere metabolites for the prebiotic control of soil-borne bacterial wilt disease. Nat. Commun. 14, 4497. 10.1038/s41467-023-40184-2 37495619 PMC10372070

[B8] Yang S. Liu H. Xie P. Wen T. Shen Q. Yuan J.. (2023). Emerging pathways for engineering the rhizosphere microbiome for optimal plant health. J. Agric. Food Chem. 71, 4441. 10.1021/acs.jafc.2c08758 36890647

[B9] Zhang C. Liu G. Xue S. Xiao L. (2013). Effect of different vegetation types on the rhizosphere soil microbial community structure in the loess plateau of China. J. Integr. Agr. 12, 2103–2113. 10.1016/S2095-3119(13)60396-2

